# The Protein Tyrosine Phosphatase Rptpζ Suppresses Osteosarcoma Development in *Trp53*-Heterozygous Mice

**DOI:** 10.1371/journal.pone.0137745

**Published:** 2015-09-11

**Authors:** Christina Baldauf, Anke Jeschke, Vincent Kanbach, Philip Catala-Lehnen, Daniel Baumhoer, Helwe Gerull, Sophia Buhs, Michael Amling, Peter Nollau, Sheila Harroch, Thorsten Schinke

**Affiliations:** 1 Department of Osteology and Biomechanics, University Medical Center Hamburg Eppendorf, Hamburg 20246, Germany; 2 Bone Tumor Reference Center at the Institute of Pathology, University Hospital Basel, Basel 4031, Switzerland; 3 Research Institute Children’s Cancer Center and Clinic of Pediatric Hematology and Oncology, University Medical Center Hamburg Eppendorf, Hamburg, Hamburg 20246, Germany; 4 Department of Neuroscience, Institute Pasteur, Paris 75624, France; Université de Lyon—Université Jean Monnet, FRANCE

## Abstract

Osteosarcoma (OS), a highly aggressive primary bone tumor, belongs to the most common solid tumors in growing children. Since specific molecular targets for OS treatment remain to be identified, surgical resection combined with multimodal (neo-)adjuvant chemotherapy is still the only way to help respective individuals. We have previously identified the protein tyrosine phosphatase Rptpζ as a marker of terminally differentiated osteoblasts, which negatively regulates their proliferation *in vitro*. Here we have addressed the question if Rptpζ can function as a tumor suppressor protein inhibiting OS development *in vivo*. We therefore analyzed the skeletal phenotype of mice lacking *Ptprz1*, the gene encoding Rptpζ on a tumor-prone genetic background, i.e. *Trp53*-heterozygosity. By screening a large number of 52 week old *Trp53*-heterozygous mice by contact radiography we found that *Ptprz1*-deficiency significantly enhanced OS development with 19% of the mice being affected. The tumors in *Ptprz1*-deficient *Trp53*-heterozygous mice were present in different locations (spine, long bones, ribs), and their OS nature was confirmed by undecalcified histology. Likewise, cell lines derived from the tumors were able to undergo osteogenic differentiation *ex vivo*. A comparison between *Ptprz1*-heterozygous and *Ptprz1*-deficient cultures further revealed that the latter ones displayed increased proliferation, a higher abundance of tyrosine-phosphorylated proteins and resistance towards the influence of the growth factor Midkine. Our findings underscore the relevance of Rptpζ as an attenuator of proliferation in differentiated osteoblasts and raise the possibility that activating Rptpζ-dependent signaling could specifically target osteoblastic tumor cells.

## Introduction

Although OS represents the most prevalent primary bone tumor, its incidence is rather low, with less than 1:100.000 [1–3]. Nevertheless, since children are most commonly affected, OS belongs to the leading causes of cancer-related death within the pediatric age group, especially since the tumors are typically very aggressive with the ability to metastasize. This explains why a molecular understanding of OS development is highly relevant, also because conventional chemotherapy following surgical resection only leads to a 5-year survival rate below 70% [4–6]. The general concept of tumorigenesis, i.e. an accumulation of gene mutations affecting oncogenes and tumor suppressor genes, is most likely also valid for OS development, where the mesenchymal osteoprogenitors transform into a state of unlimited proliferation and subsequent bone formation [7,8]. Therefore, many attempts have been made to identify OS-relevant genes by genome-wide expression or sequencing analysis of OS tissue samples [9–18]. Although several candidates have been identified by these approaches, the establishment of a definite gene panel is still difficult, mostly explained by the large heterogeneity and the enhanced genomic instability of OS compared to many other tumors [19–21]. With respect to OS susceptibility, some loci were only recently identified by candidate or genome-wide association studies in large cohorts [22–25]. Moreover, the previously performed genetic analysis of individuals with inherited disorders characterized by high OS incidence has demonstrated that mutations of common tumor suppressor genes (*TP53*, *RB1*, *RECQL4*) also predispose to OS development [8]. One of these disorders is Li-Fraumeni syndrome, which can be caused by heterozygous germline mutations of *TP53* and which is characterized by increased cancer risk with OS development in more than 10% of the affected individuals [26–28]. Consistent with the tumor suppressor function of TP53 in OS, somatic *TP53* mutations were also identified in the majority of human OS tumors or cell lines [29–31]. Likewise, OS development was observed in mouse models with heterozygous or osteoblast-specific inactivation of *Trp53*, the murine homologue of *TP53* [32–37].

In an attempt to identify markers for terminally differentiated osteoblasts we have previously performed genome-wide expression analysis comparing murine primary calvarial osteoblasts at a non-mineralized and a mineralized stage [38]. In addition to the known markers of osteoblast differentiation, that were all regulated as expected, we identified several genes with increased expression in mineralized cultures that were not previously analyzed in the context of bone remodeling. Of note, the gene displaying the strongest level of induction in this particular experiment was *Ptprz1*, encoding the transmembrane protein tyrosine phosphatase Rptpζ [39]. Since many growth factors activate cellular proliferation by inducing tyrosine phosphorylation of intracellular signaling proteins, this finding suggested that Rptpζ could negatively regulate osteoblast differentiation, thus being responsible to induce a postmitotic state in terminally differentiated mineralizing osteoblast cultures [40]. This hypothesis was supported by *ex vivo* experiments, where we took advantage of a previously established *Ptprz1*-deficient mouse model, which does not display an obvious phenotype in a non-challenged situation [41]. Of note, although *Ptprz1*-deficient mice only displayed a moderate osteopenia at 1 year of age, primary osteoblasts derived from newborn *Ptprz1*-deficient mice had an increased proliferation rate compared to wildtype cultures [38]. Likewise, transfection of an Rptpζ expression plasmid into MC3T3-E1 osteoblasts significantly reduced their proliferation rate in a dose-dependent manner.

Although these experiments demonstrated that Rptpζ negatively regulates osteoblast proliferation, we did not observe spontaneous OS development in *Ptprz1*-deficient mice until the age of 18 months. Since it is well established however that tumor development requires an accumulation of several gene mutations, we hypothesized that a potential OS suppressor function of Rptpζ can only be uncovered in the context of an already existing mutation of a common tumor suppressor gene [42]. We therefore crossed *Ptprz1*-deficient mice into a *Trp53*-heterozygous background and monitored OS development by screening through contact Xray. Here we found that OS development in 12 month old *Trp53*-heterozygous mice was significantly enhanced by *Ptprz1*-deficiency, thereby providing evidence for a tumor suppressor function of Rptpζ.

## Materials and Methods

### 1. Expression analysis

RNA was isolated from various tissues of 6 week old mice and from primary osteoblasts at various stages of differentiation. More specifically, these cells were isolated by sequential collagenase digestion from the calvariae of 5 days old mice as described [43]. After removal of the co-purified macrophage-like cells by CD11b-immunoaffinity [44,45] cells were plated and cultured in α-MEM until they reached 80% confluency (day 0). We then added ascorbic acid (50 μg/ml) and ß-glycerophosphate (10 mM) to the cultures to induce osteogenic differentiation for 4, 7, 10, 15, 20 or 25 days. RNA isolation was performed with the RNeasyMini kit (Qiagen). For osteoclastogenesis bone marrow cells were isolated from 12 week old mice and induced to differentiate into osteoclasts by addition of 1,25-vitamin D3, M-CSF and RANKL as described [45]. We also isolated RNA from human osteoblasts (PromoCell #C-12720) and human OS cell lines (SaOS-2, ATCC #HTB-85; U2-OS, ATCC #HTB-96), all of them differentiated for 0, 7 or 15 days by using the commercially available osteoblast mineralization medium (PromoCell #C27020) or by addition of ascorbic acid (50 μg/ml), ß-glycerophosphate (10 mM) and dexamethasone (100 nM) to the culture medium. Concentration and quality of RNA were measured using a NanoDrop ND-1000 system (NanoDrop Technology). For qRT-PCR expression analysis, 1 μg of RNA was reversed transcribed using SuperScriptIII (Invitrogen) according to manufacturer’s instructions. Reactions were performed using predesigned TaqMan gene expression assays (Applied Biosystems) with *Gapdh* as internal control. We additionally isolated RNA from CD11b-negative osteoblast cultures at day 5 and day 12 of differentiation to perform genome-wide expression analysis using Affymetrix Gene Chips (Affymetrix MG 430 2.0). The respective data sets have been deposited in GEO under accession code GSE71565.

### 2. Mouse models

*Trp53*-deficient mice were purchased from the Jackson Laboratories (#002101). Their genotyping was performed with the primers 5´-ACA GCG TGG TGG TAC CTT AT-3´, 5´-TAT ACT CAG AGC CGG CCT-3´, and 5´-CTA TCA GGA CAT AGC GTT GG-3´, giving rise to a 450 bp and a 650 bp fragment for the wildtype and mutant allele, respectively. *Ptprz1*-deficient mice have been described previously [38]. Their genotyping was performed with the primers 5´-AGA TCC ATT CGT CTT GCA GCC TCC-3´, 5´-CAC CTG CCT GGA AAA CTT GTA CTG-3´, 5´-GAA AAG CGC CTC CCC TAC CCG GTA GAA TTG AC-3´ and 5´-CCA GAC ATG ACA CCC CAA TGC CTG AAC ATC TC-3´, giving rise to a 400 bp and a 650 bp fragment for the wildtype and mutant allele, respectively. To exclude any possible influence of genetic background, all analyses were performed with littermates obtained from compound heterozygous matings.

### 3. Skeletal analysis

After sacrifice all skeletons were immediately analyzed by contact radiography using a Faxitron Xray cabinet (Faxitron Xray Corp.). For further analysis the dissected skeletons were fixed in 3.7% PBS-buffered formaldehyde for 18 hours at 4°C, before they were stored in 80% ethanol. μCT analysis was performed with a μCT 40 (Scanco Medical). For histology, specific skeletal elements were dehydrated in ascending alcohol concentrations and then embedded in methylmetacrylate as described previously [43]. Sections of 5 μm thickness were cut in the sagittal plane on a Microtec rotation microtome (Techno-Med GmbH). All sections were stained by von Kossa/van Gieson or toluidine blue staining procedures as described [43].

### 4. Characterization of murine OS cell lines

For *ex vivo* OS cell culture, tumor tissues were dissected and cleaned in PBS containing antibiotics. Tumors were then minced with a scissor and placed into a culture dish in the presence of α-MEM (including 10% FBS and 1% Penicillin/Streptomycin). When the outgrowing cells reached 80–90% confluency, cells were trypsinized and plated onto new culture dishes without tissue residues. OS cell lines were established by repeated passaging (more than 12 times) under regular tissue culture conditions. Osteogenic differentiation was induced by adding ascorbic acid (50 μg/ml) and ß-glycerophosphate (10 mM) to the cultures. After 10 and 20 days we assessed matrix mineralization by alizarin red incorporation as described [45]. To monitor the proliferation rate cells were seeded in triplicate cultures at an initial density of 20.000 cells per well of a 6-well-plate and counted every day using a Neubauer chamber. Additionally we measured BrdU incorporation using the Biotrak Cell Proliferation Kit (Amersham) according to the manufacturer's instructions. To assess the proliferative behavior in the presence of Midkine (Mdk) we performed short-term treatment with increasing concentrations of murine recombinant Mdk (Peprotech) in serum-free medium. After 12 hours of incubation BrdU incorporation was determined as described above. The effects of long-term administration were analyzed by monitoring cell growth for seven days in the presence or absence of 100 ng/ml Mdk.

### 5. Far Western Blotting with SH2-domains

OS cell lines were deprived of serum for 5 hours in α-MEM and re-stimulated with 10% FBS for 5 minutes. For whole cellular protein extraction cells were washed two times with ice-cold PBS and immediately lysed in KLB-buffer (150 mN NaCl, 25 mM TrisHCL (pH 7.4), 5 mM EDTA, 1% Triton X-100, 10% glycerol, 10 mM sodium pyrophosphate, 1 mM sodium ortho-vanadate, 10 mM ß-glycerolphosphate, 1 mM PMSF, 0.2 mg/ml of aprotinin (Sigma), 10 mM NaF, 0.1 mM freshly prepared sodium pervanadate and 1 mM DTT). After incubation on ice for 30 min cellular lysates were cleared by centrifugation at 4°C for 10 min and supernatants were stored at -80°C. SH2 profiling based on far-Western blotting was essentially performed as described previously [46,47]. Briefly, 15 μg of OS lysates were separated by SDS-PAGE (4–12% acrylamide gels) and transferred to PVDF membranes. After blocking (10% skim milk in TBST-buffer), replicate blots were separately probed with 1 μg/ml of biontinylated SH2 domains precomplexed with streptavidin-horseradish peroxidase. Signals were visualized by chemoluminescence using ECL blotting reagent system (Amersham).

### 6. Site-directed mutagenesis and DNA transfection in human OS cell lines

To determine the function of Rptpζ as a negative regulator of osteoblast proliferation, we introduced two mutations into the previously described Rptpζ expression plasmid [38]. The first mutation (NM_001206838.1:exon21:c.G3218T:p.C1073S) is located in the active site of Rptpζ and should destroy its ability to desphosphorylate specific substrates [48]. The second mutation (NM_001206838.1:exon22:c.G3227T:p.G1076V) was identified by analysis of exome sequencing data sets from 17 human OS samples. Both mutations were introduced using the QuikChange Lightning Multi Site-Directed Mutagenesis Kit (Stratagene) according to manufacturer’s instructions. For transfections, the human SaOS-2 and U2-OS cells were seeded into 96-well-plates at an initial density of 1000 cells per well. Cells were cultured to 50–70% confluence and transfected with 0.5 μg of wildtype and/or mutant Rptpζ expression plasmids using the Lipofectamine™ 2000 reagent (Invitrogen) according to manufacturer’s instructions. 48 h after transfection BrdU incorporation was measured as described above.

### 7. Statistical analysis

All data presented in the manuscript were obtained from the analysis of littermates (n ≥ 5) and are presented as means ± standard deviations. Statistical analyses were performed by by the method of Kruskal and Wallis followed by Dunn´s post-test, by Fishers`s exact test, by one-way or two-way ANOVA followed by Dunnett’s or Bonferroni’s post-test (after estbalishing normality and homoscedasticity), or by unpaired, two-tailed Student’s *t*-test, as indicated in the figure legends, using the commercial software GraphPad Prism5 (version 5.02). P-values below 0.05 were considered statistically significant.

### 8. Ethics Statement

All animal experiments were approved by the animal facility of the University Medical Center Hamburg Eppendorf and by the “Amt für Gesundheit und Verbraucherschutz” (Org529).

## Results

**Fig 1:**
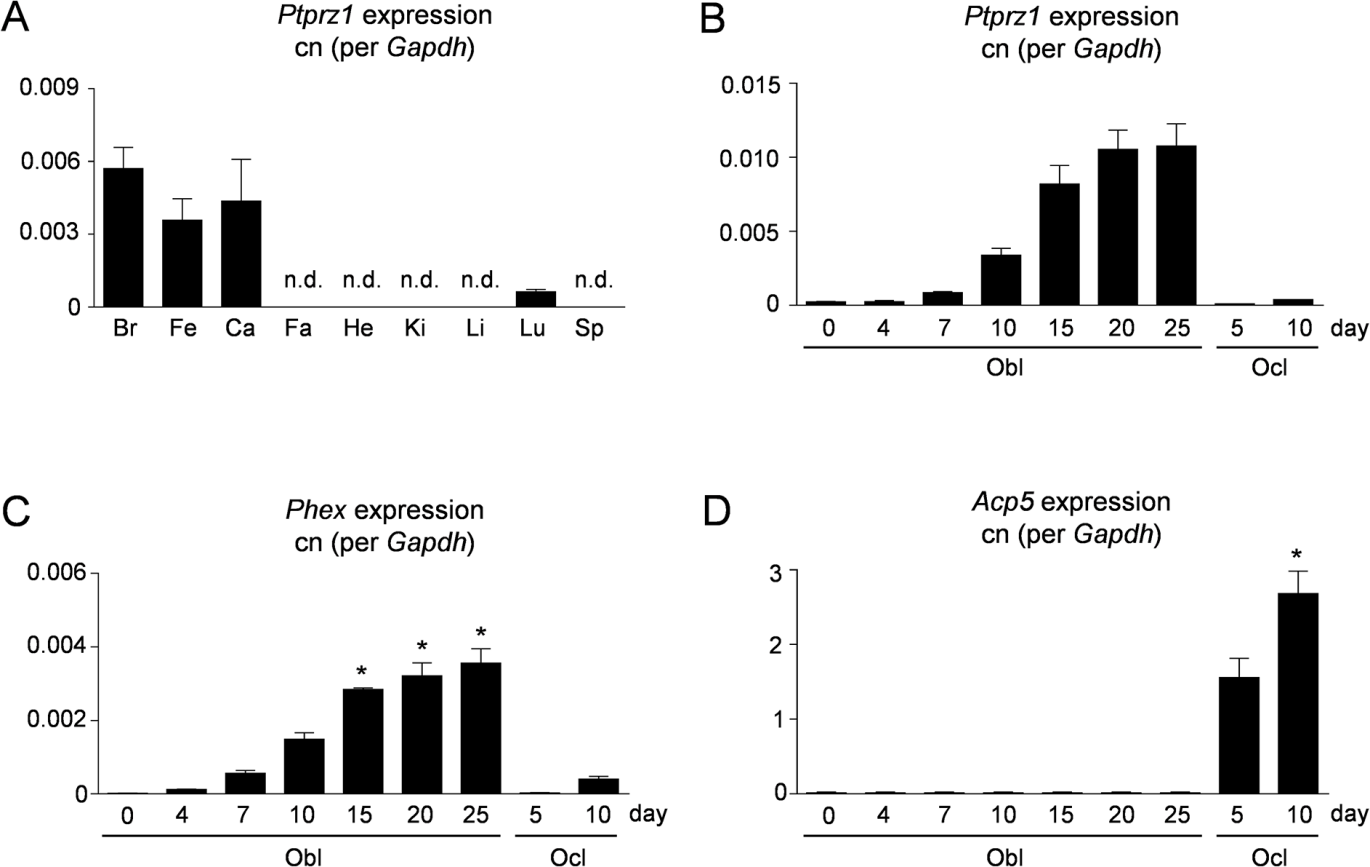
*Ptprz1* expression by differentiated osteoblasts. (A) qRT-PCR monitoring *Ptprz1* expression in brain (Br), femur (Fe), calvaria (Ca), fat (Fa), heart (He), kidney (Ki), liver (Li), lung (Lu) and spleen (Sp). (B) qRT-PCR monitoring *Ptprz1* expression in primary osteoblasts (Obl) or osteoclasts (Ocl) at different stages of differentiation. Bars represent mean ± SD (n = 3). (C) qRT-PCR monitoring expression of the osteocyte marker *Phex*. (D) qRT-PCR monitoring expression of the osteoclast marker *Acp5* (encoding TRAP). Values represent copy number (cn) relative to *Gadph*. Bars represent mean ± SD (n = 3). Asterisks indicate statistical significance vs. Obl day 0 (p<0.05, Kruskal-Wallis followed by Dunn’s post-test).

Since our previously published genome-wide expression analysis was performed with primary murine osteoblasts still containing the co-purifying macrophage population, we first analyzed, if *Ptprz1* is also differentially expressed in primary osteoblast cultures after depletion of the macrophage population by CD11b immunoaffinity [44]. For that purpose a comparative genome-wide expression analysis was performed with CD11b-negative osteoblasts at day 5 and day 12 of differentiation. By sorting all genes according to the logarithmic ratio of signal intensity (SLR, signal log ratio) we found that the expression of well-established osteoblast markers increased during the course of differentiation (Table 1). Importantly, the same was the case for *Ptprz1*, whereas other genes encoding protein tyrosine phosphatases were not differentially expressed to the same extent (Table 2). We next monitored *Ptprz1* expression in various tissues and primary bone cells from wildtype mice by qRT-PCR. Among tissues we detected the highest expression levels in brain, as well as in femoral and calvarial bone (Fig 1A). *Ptprz1* expression was also detectable in lung, but not in fat, heart, kidney, liver and spleen. To identify the primary bone cell type expressing *Ptprz1* we next analyzed primary calvarial osteoblasts and primary bone marrow-derived osteoclasts at different stages of differentiation. Here we found that *Ptprz1* expression progressively increased during the course of osteoblast differentiation, while expression in osteoclast cultures was barely detectable (Fig 1B–1D). These data demonstrate that *Ptprz1* represents an osteoblast differentiation marker, whose expression is restricted to few tissues.

**Table 1:** Genes encoding osteogenesis markers displaying differential expression during primary osteoblast differentiation. Given are the Affymetrix signal intensities at two stages of differentiation and the signal log ratios (SLR).

Gene	day 5	day 12	SLR
*Mepe*	1.5	301.8	7.0
*Dkk1*	14.5	685.1	5.6
*Ibsp*	157.8	5959.9	5.0
*Dmp1*	154	3103.2	4.4
*Phex*	35.6	554.7	3.9
*Ifitm5*	186.0	2244.3	3.7
*Bglap*	646.7	6611.7	3.5

**Table 2:** Genes encoding PTPs displaying differential expression during primary osteoblast differentiation. Given are the Affymetrix signal intensities at two stages of differentiation and the signal log ratios (SLR).

Gene	day 5	day 12	SLR
*Ptprz1*	18.7	590.3	5.1
*Ptpro*	15.7	54.2	1.8
*Ptprc*	60.9	134.1	1.2
*Ptpn18*	47.0	14.1	1.2
*Ptpn20*	25.9	64.6	1.2
*Ptprt*	5.2	10.7	1.1
*Ptpn6*	42.2	53.4	0.9
*Ptpn2*	1219.1	1735.0	0.4

As we have previously established that Rptpζ negatively regulates osteoblast proliferation *in vitro* [38], we next addressed the question, whether its inactivation would trigger OS development. Since *Ptprz1*-deficiency alone does not result in the presence of OS (until the age of 18 months), we first generated mice lacking both, *Ptprz1* and *Trp53*, and compared them to *Trp53-*deficient littermates. Given the early lethality associated with *Trp53-*deficiency, mostly explained by lymphomas [32], we performed the skeletal analysis at 12 weeks of age. Using contact Xray we did not detect any signs of OS development in *Trp53-*deficient mice, regardless of the presence or absence of *Ptprz1* (S1 Fig). The same was true for undecalcified sections from the spine or the tibia of the corresponding mice.

**Fig 2:**
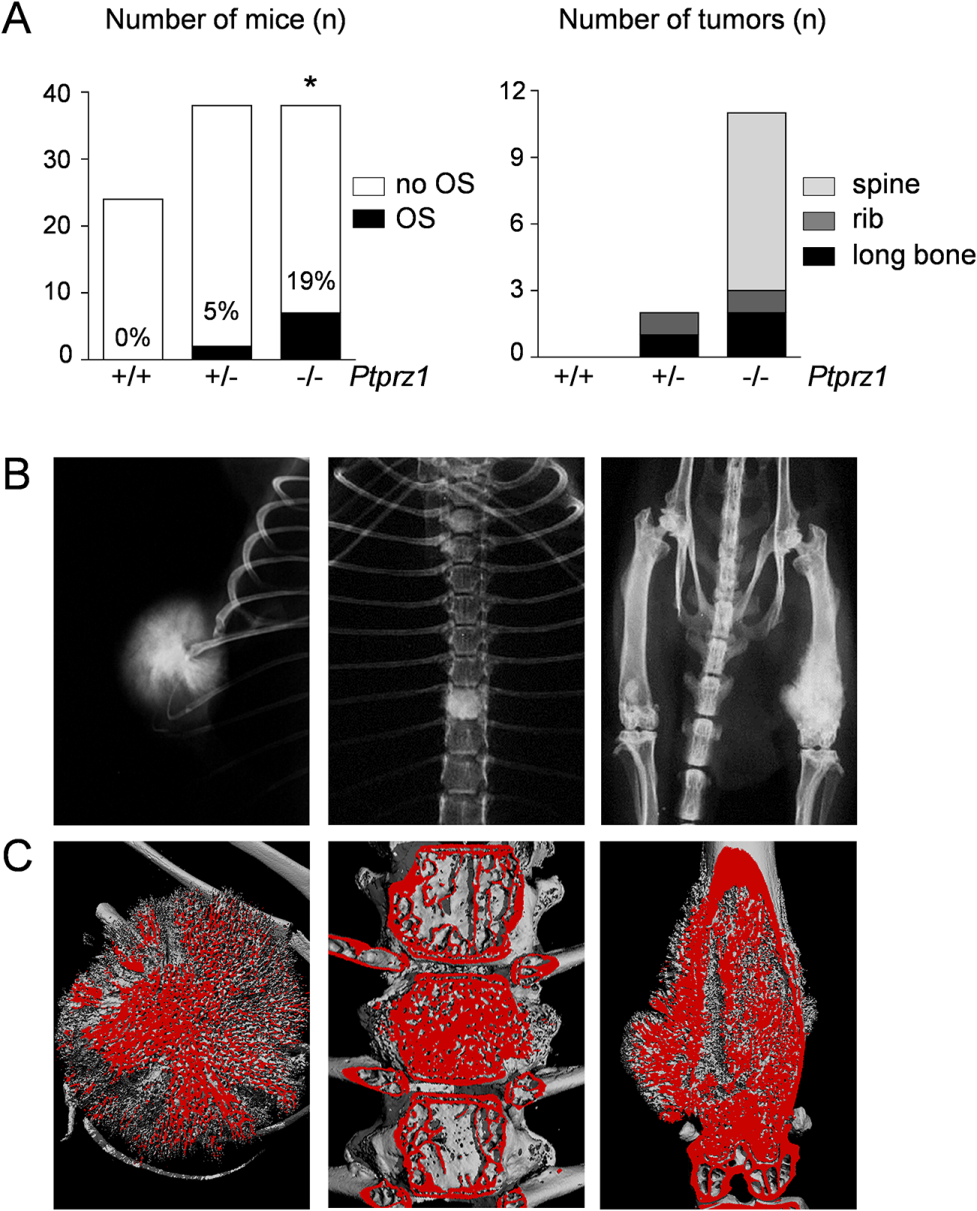
Skeletal tumors in *Ptprz1*-deficient *Trp53*-heterozygous mice. (A) OS development assessed by screening of 12 month old *Trp53*-heterozygous mice with the indicated *Ptprz1* genotypes. The left panel shows the number of analyzed mice (white bars) and the percentage of mice with OS (black bars). The asterisk indicates statistical significance vs. *Trp53* ^*+/-*^ */Ptprz1* ^*+/+*^ (p<0.05, two-tailed Fishers`s exact test). The right panel shows the total number of tumors and their location. (B) Representative contact Xrays from *Ptprz1*-deficient *Trp53*-heterozygous mice with OS in the three different locations. (C) μCT images from the same tumors.

Since *Trp53-*heterozygous mice display reduced lymphoma development and therefore increased life span, we went on to generate *Ptprz1*-deficient *Trp53-*heterozygous mice (*Trp53* ^*+/-*^ */Ptprz1* ^*-/-*^) and compared them to *Trp53-*heterozygous mice (*Trp53* ^*+/-*^ */Ptprz1* ^*+/+*^) and to *Trp53-*heterozygous mice lacking one *Ptprz1* allele (*Trp53* ^*+/-*^ */Ptprz1* ^*+/-*^). All animals were analyzed at 52 weeks of age using contact Xray to screen for the presence of OS, unless OS development was observed earlier (S1 Table). While none of the 24 *Trp53* ^*+/-*^ */Ptprz1* ^*+/+*^ mice displayed obvious skeletal tumors, OS development was detected in 7 out of 38 *Trp53* ^*+/-*^ */Ptprz1* ^*-/-*^ mice (Fig 2A). In *Trp53* ^*+/-*^ */Ptprz1* ^*+/-*^ mice skeletal tumors were visible in 2 out of the 38 mice that were analyzed. The skeletal tumors found in *Trp53* ^*+/-*^ */Ptprz1* ^*-/-*^ and *Trp53* ^*+/-*^ */Ptprz1* ^*+/-*^ mice were present in three different locations (Fig 2B), i.e. ribs, spine or long bones, but not in craniofacial bones, and μCT analysis demonstrated that they consisted of mineralized tissue (Fig 2C). Taken together, these data provide strong evidence for a tumor suppressor function of Rptpζ, as the OS incidence in 52 week old *Trp53-*heterozygous mice was raised from 0% to 19% by *Ptprz1*-deficiency.

**Fig 3:**
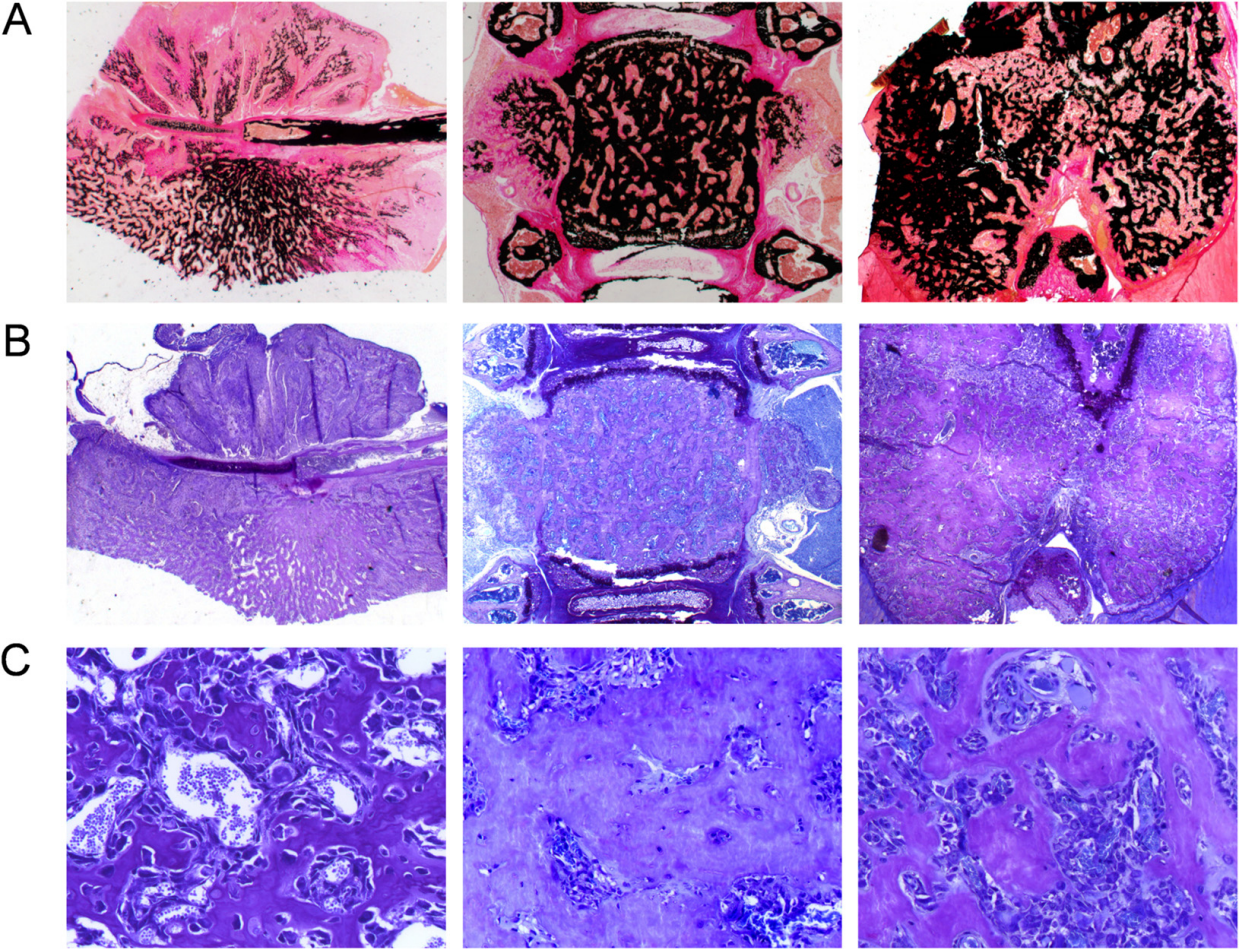
OS nature of skeletal tumors in *Ptprz1*-deficient *Trp53*-heterozygous mice. (A) Von Kossa/Van Gieson staining of undecalficied sections confirms that the tumors contain mineralized matrix (stained black). (B) Toluidine blue staining demonstrating dark blue staining of cartilage areas and light blue staining of the tumors. (C) Higher magnification images reveal that the tumors represented bony tissue with osteocytes embedded into the mineralized matrix.

**Fig 4:**
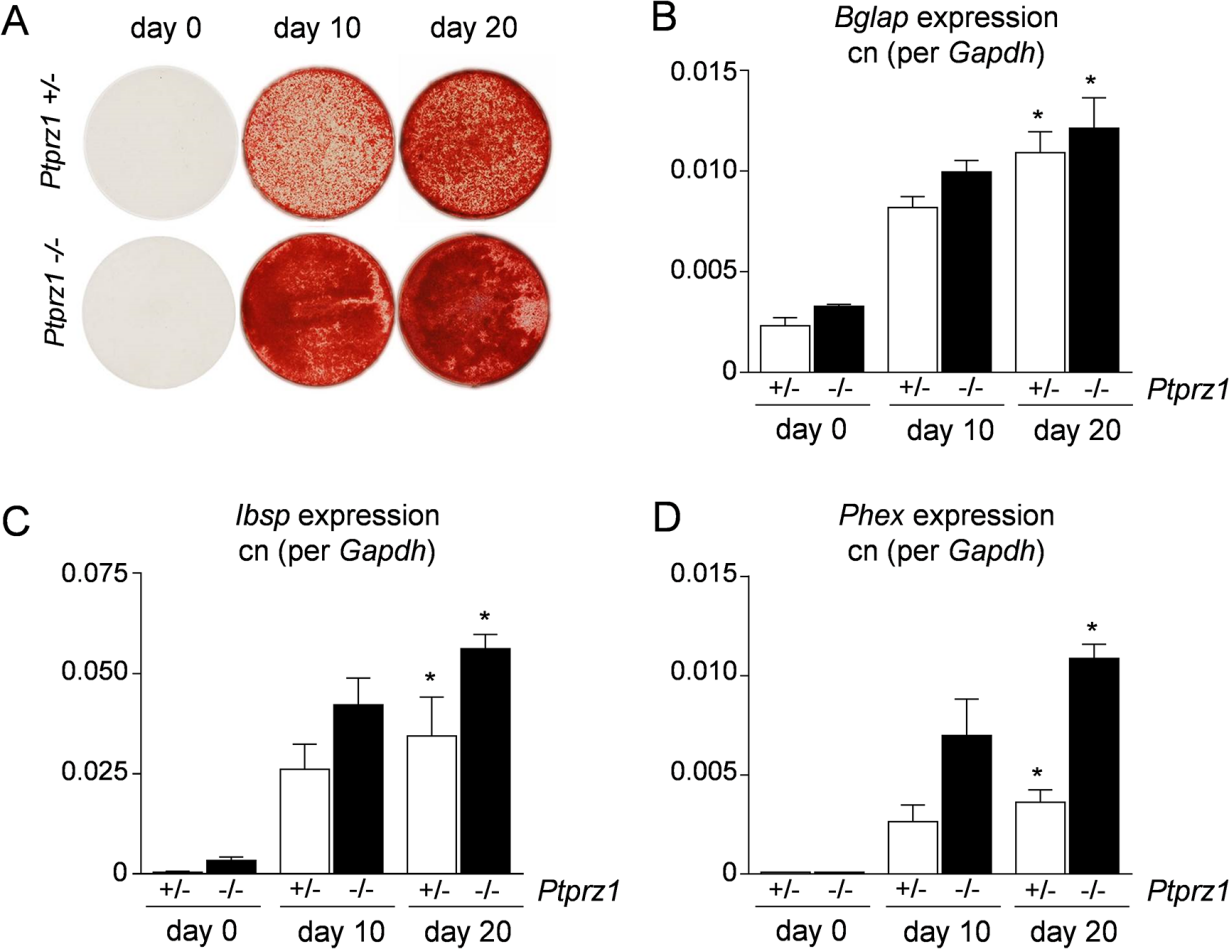
Osteogenic differentiation of OS cell lines. (A) Alizarin red staining of cells derived from *Trp53*-heterozygous mice with either one *Ptprz1* allele (+/-) or with *Ptprz1*-deficiency (-/-) reveals that both cell lines are able to form a mineralized matrix after 10 and 20 days of differentiation induced by ascorbic acid and ß-glycerophosphate. (B-D) qRT-PCR expression analysis shows that the differentiation is accompanied by increased expression of *Bglap* (encoding Osteocalcin), *Ibsp* (encoding Bone sialoprotein) and *Phex*. Values represent copy number (cn) relative to *Gadph*. Bars represent mean ± SD (n = 3). Asterisks indicate significant differences towards day 0 of corresponding genotype (p<0.05, Kruskal-Wallis followed by Dunn’s post-test).

To confirm that the tumors represented OS we further applied undecalcified histology (Fig 3A). By toluidine blue staining of proteoglycans we were able to rule out that the tumors are chondrogenic (Fig 3B), which was also supported by the fact, that their mineralized matrix contained osteocytes (Fig 3C). We additionally isolated OS cell lines from two different tumors (*Trp53* ^*+/-*^ */Ptprz1* ^*+/-*^ and *Trp53* ^*+/-*^ */Ptprz1* ^*-/-*^) and cultured them in the presence of ascorbic acid and ß-glycerophosphate. Here we found that both cell lines were able to form a mineralized matrix *ex vivo* (Fig 4A). Moreover, they both displayed differential expression of osteoblast differentiation markers (Fig 4B–4D), thereby confirming the OS nature of the tumors.

**Fig 5:**
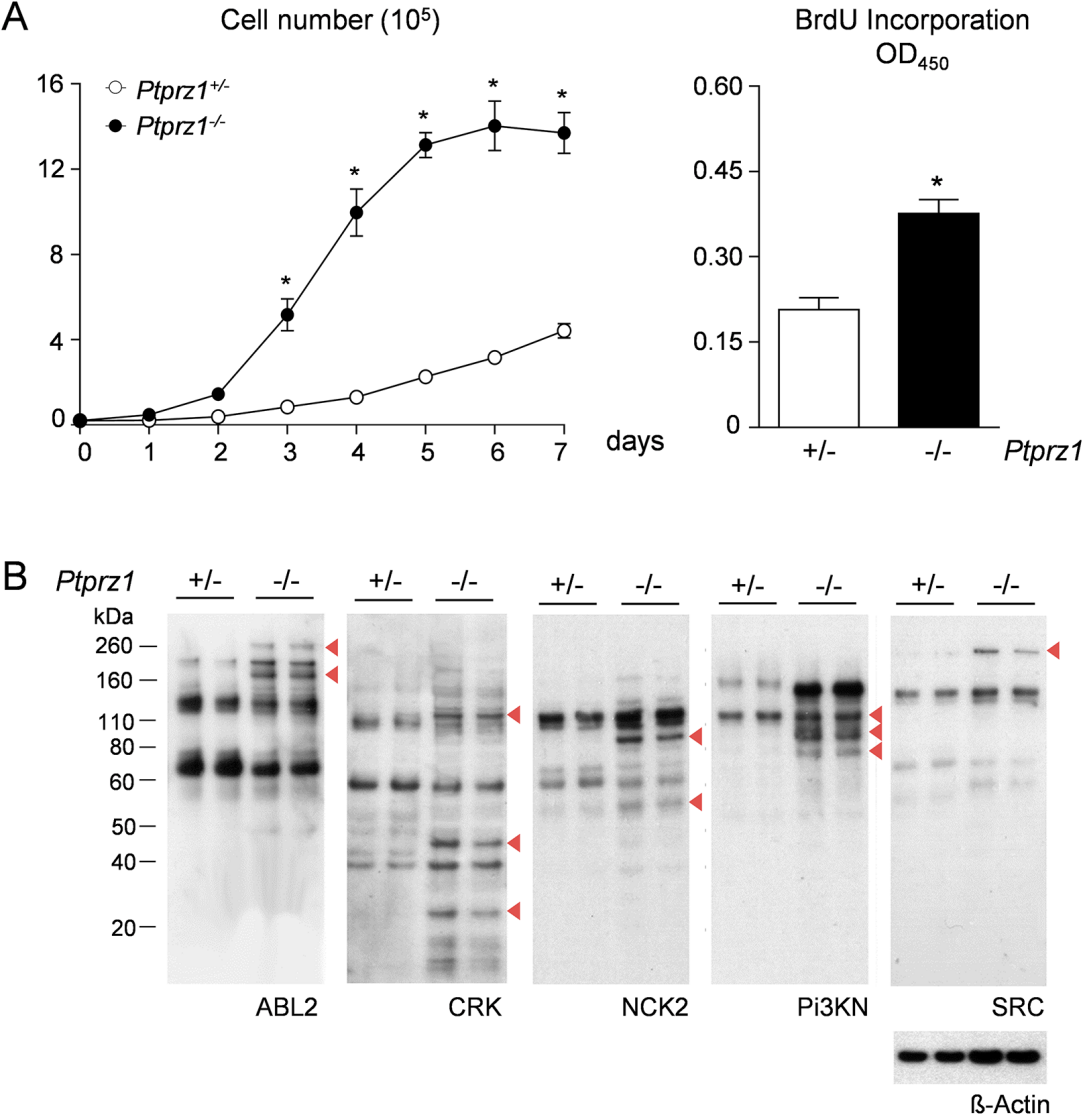
Proliferation and tyrosine phosphorylation in OS cell lines. (A) Proliferative capacity of tumor cells derived from *Trp53*-heterozygous mice with either one *Ptprz1* allele (+/-) or with *Ptprz1*-deficiency (-/-). The growth curves (left) and the BrdU incorporation assays (right) demonstrate increased proliferation in the cases of *Ptprz1*-deficiency. Bars represent mean ± SD (n≥3). Asterisks indicate significant differences between the two genotypes (p<0.05, two-way ANOVA followed by Bonferroni’s post-test (left panel) or two-tailed Student’s *t*-test (right panel)). (B) SH2 profiling with different SH2 domains reveals differences in tyrosine phosphorylation of specific proteins (indicated by arrowheads). Re-probing of stripped membranes with anti ß-actin mAb served as control for equal loading. Analyses were performed in duplicate with cell extracts harvested at different cell densities.

**Fig 6:**
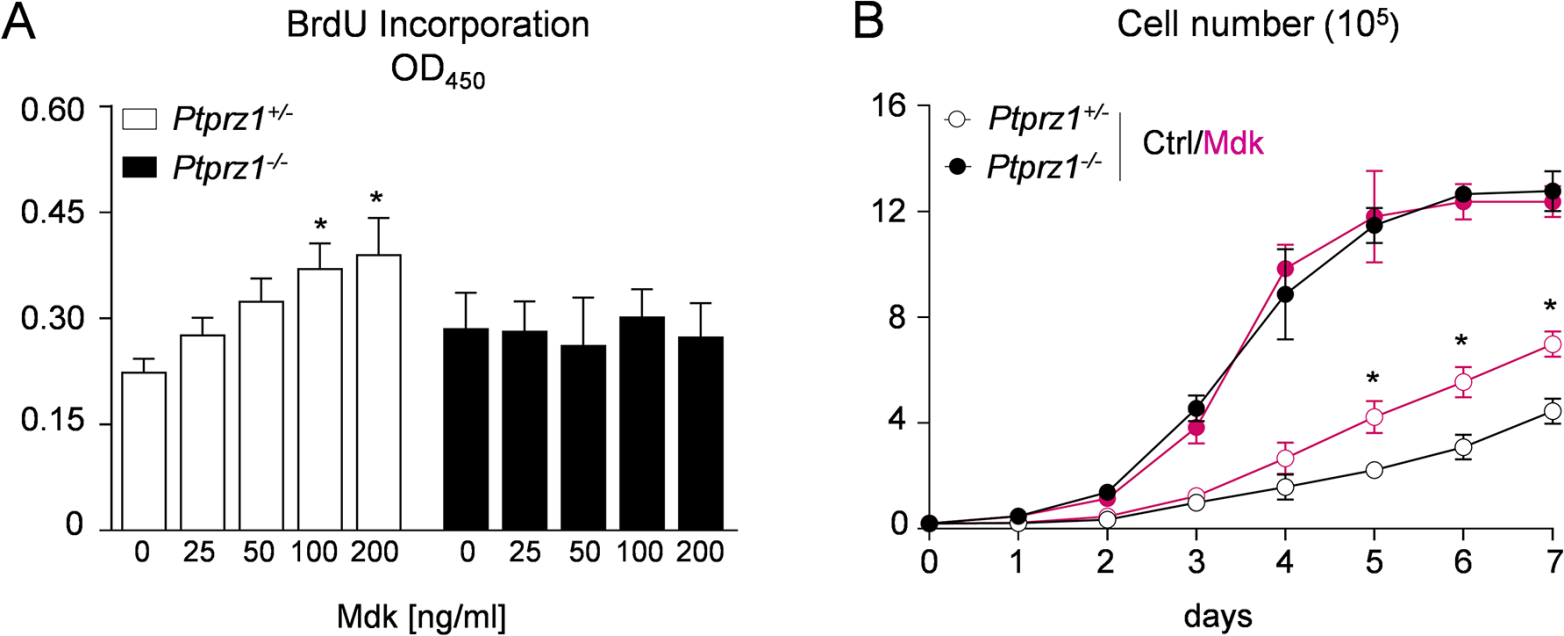
Effects of Mdk on proliferation of OS cell lines. (A) BrdU incorporation assays with the indicated OS cell lines performed in the presence of Mdk at different concentrations. Bars represent mean ± SD (n = 8). Asterisks indicate significant differences towards controls without Mdk (p<0.05, one-way ANOVA followed by Dunnett’s post-test). (B) Growth curves of OS cell lines in the presence or absence of Mdk (100 ng/ml). Asterisks indicate significant differences towards controls of *Ptprz1* ^*+/-*^ cells without Mdk (p<0.05, two-way ANOVA followed by Bonferroni’s post-test).

We additionally compared the *ex vivo* behaviour of the *Trp53* ^*+/-*^ */Ptprz1* ^*+/-*^ and *Trp53* ^*+/-*^ */Ptprz1* ^*-/-*^ OS cell lines with respect to proliferation and tyrosine phosphorylation status. We observed that the cell line deficient in Rptpζ proliferated at higher rate, which was also confirmed by BrdU incorporation assays (Fig 5A). We further assessed differences in the state of tyrosine phosphorylation between the *Trp53* ^*+/-*^ */Ptprz1* ^*+/-*^ and *Trp53* ^*+/-*^ */Ptprz1* ^*-/-*^ OS cell lines using far-Western Blotting with different SH2-domains [46,47]. Here we observed that several proteins were specifically detected in *Trp53* ^*+/-*^ */Ptprz1* ^*-/-*^ cells with the most obvious differences in the case of SH2-domains of ABL2, CRK, NCK2, Pi3KN and SRC (Fig 5B, S2 Fig). In addition, we analyzed the response of both cell lines to the heparin-binding growth factor Mdk, which has been shown to antagonize Rptpζ in other cell types [49,50]. Using BrdU incorporation assays we found that Mdk increased the proliferation in *Trp53* ^*+/-*^ */Ptprz1* ^*+/-*^ cells in a dose-dependent manner, whereas *Trp53* ^*+/-*^ */Ptprz1* ^*-/-*^ cells did not respond (Fig 6A). Likewise, long-term administration of Mdk for 7 days significantly enhanced cellular growth only in *Trp53* ^*+/-*^ */Ptprz1* ^*+/-*^ cells (Fig 6B). These data demonstrate that the pro-proliferative influence of Mdk on OS cell lines depends on Rptpζ interaction, which is potentially relevant, as *MDK* was found over-expressed in human OS [51,52].

**Fig 7:**
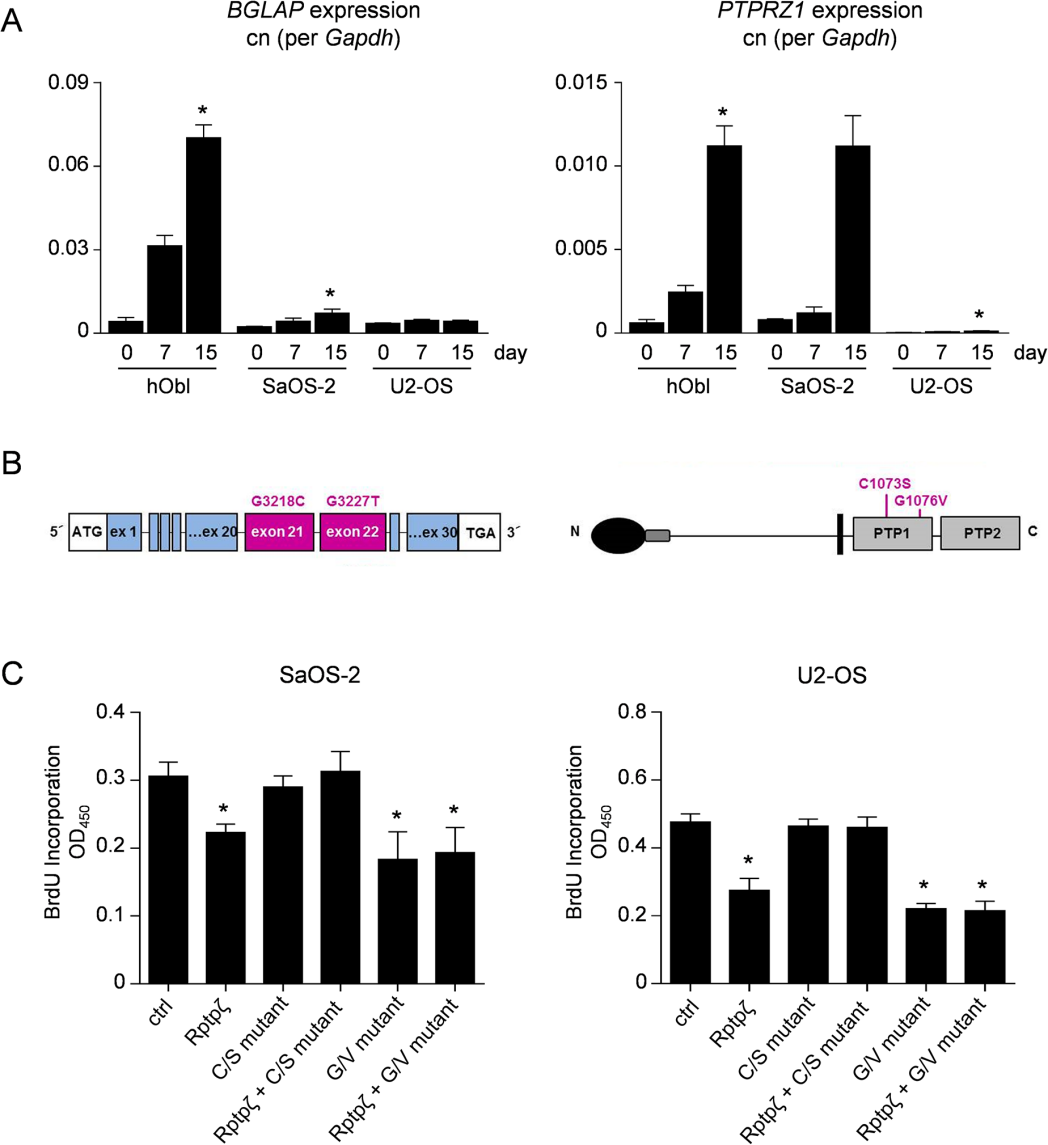
Functional analysis of Rptpζ mutations. (A) qRT-PCR monitoring expression of *BGLAP* (left) and *PTPRZ1* (right) in human primary osteoblasts (hObl), SaOS-2 or U2-OS cells at different stages of differentiation. Values represent copy number (cn) relative to *GAPDH*. Bars represent mean ± SD (n = 3). Asterisks indicate statistical significance vs. day 0 (p<0.05, Kruskal-Wallis followed by Dunn’s post-test). (B) Schematic presentation of the *PTPRZ1* gene and Rptpζ protein showing the location of the two mutations that have been introduced into a Rptpζ expression plasmid. (C) BrdU incorporation assay with SaOS-2 and U2-OS cells after transfection of wildtype and/or mutant Rptpζ expression plasmids as indicated. Bars represent mean ± SD (n = 6). Asterisks indicate significant differences towards cells transfected with empty vector (ctrl) (p<0.05, one-way ANOVA followed by Dunnett’s post-test).

To address the question if Rptpζ would act as a tumor suppressor in human OS we first analyzed, if *PTPRZ1* is differentially expressed during differentiation of human osteoblasts. We therefore induced osteogenic differentiation of commercially available primary osteoblasts and of two OS cell lines (SaOS-2 and U2-OS). Here we found that *PTPRZ1* expression was increased compared to non-differentiated cells after 15 days in all three cell types, thereby confirming its differential expression in human osteoblasts (Fig 7A). We next analyzed exome sequencing data sets from 17 human OS samples, thereby identifying a heterozygous germline mutation (G3227T) in exon 22 of the *PTPRZ1* gene in one of the cases. Since the mutation causes an amino acid substitution (G1076V) within the first protein tyrosine phosphatase domain, we hypothesized that it might interfere with Rptpζ activity. To address this possibility we introduced the corresponding mutation into an Rptpζ expression plasmid [38], and we additionally introduced another mutation (C1073S) within the active site of Rptpζ, serving as a control (Fig 7B). The mutant plasmids were then transfected into non-differentiated SaOS-2 and U2-OS cells, either alone or in combination with intact Rptpζ expression plasmid, before assessing proliferation by BrdU incorporation assays. In both cell lines we found that Rptpζ significantly reduces BrdU incorporation, thereby confirming its role as an attenuator of proliferation also in human osteoblasts (Fig 7C). As expected, the anti-proliferative activity of Rptpζ was fully abolished by the C1073S mutation, which also acted in a dominant negative fashion. Most importantly however, introducing the G1076V mutation did not interfere with the anti-proliferative function of Rptpζ, thereby essentially ruling out its contribution to OS development in the identified individual carrying this mutation in a heterozygous state.

## Discussion

Rptpζ, encoded by the *Ptprz1* gene, is primarily expressed in the central nervous system, mostly by astrocytes and oligodendrocytes [53]. Although *Ptprz1*-deficient mice do not display an obvious phenotype in a non-challenged situation, they were found to have impaired recovery from demyelinating lesions, supporting a role of Rptpζ in oligodendrogenesis [54]. In its full-length form Rptpζ represents a transmembrane protein with two intracellularly located protein tyrosine phosphatase domains [55]. The extracellular part consists of an N-terminal carbonic anhydrase-like domain followed by a fibronectin type III repeat and a large intervening sequence. Importantly, the latter domain is posttranslationally modified by glycoaminglycan attachment, and a soluble form of Rptpζ, also known as phosphacan, lacking the transmembrane and tyrosine phosphatase domains, represents one of the most abundant proteoglycans in the brain [56,57]. With respect to Rptpζ-specific ligands it was found that two heparin-binding growth factors, Pleiotrophin and Mdk, interact with the glycosaminoglycan structures of Rptpζ, thereby inhibiting the tyrosine phoshatase activity of Rptpζ [49,50,58,59]. In terms of OS development, the Mdk/Rptpζ interaction appears particularly interesting, since *MDK* was found over-expressed in human OS and positively regulated proliferation of human OS cell lines [51,52]. With respect to a possible tumor suppressor function of Rptpζ it was recently shown that its shRNA-mediated repression in prostate cancer cell lines did not only increase the migratory behavior of these cells *in vitro*, but also enhanced their metastatic potential after injection into nude mice [60]. The putative role of Rptpζ as a suppressor of OS development however, has not been studied so far.

Since we identified *Ptprz1* as the only PTP-encoding gene displaying robust differential expression during primary osteoblast differentiation, where it is required to inhibit proliferation, we addressed the question, if *Ptprz1*-deficiency would increase OS incidence on a tumor-prone genetic background. Since we did not observe OS development in 12 week old mice lacking *Trp53* and *Ptprz1*, we analyzed OS development in *Trp53*-heterozygous mice, which were monitored by Xray analysis at 12 months of age. One limitation of this screening approach was that we did not necessarily pick up smaller foci with OS characteristics in specific skeletal elements. We therefore cannot fully rule out that such transformation processes occurred in a subset of *Trp53*-heterozygous mice, as it has been reported previously [45]. On the other hand, the Xray screening allowed us to analyze the full skeletons from a large number of animals, and it was the purpose of our study to detect larger tumors, similar to the previously analyzed mouse model over-expressing the cFos proto-oncogene [61,62]. Especially since our approach immediately identified increased OS susceptibility in *Ptprz1*-deficient *Trp53*-heterozygous mice, we did not change our strategy and went on to characterize the tumors by undecalficied histology and *ex vivo* assays. Using these methods we could demonstrate that *Ptprz1*-deficiency raised the OS incidence in 52 weeks old *Trp53-*heterozygous mice from 0% to 19%, thereby providing evidence for a tumor suppressor function of Rptpζ, at least in mice.

To address the question, if Rptpζ would also act as a tumor suppressor in human OS, we first analyzed copy number data sets (CytoScan Arrays) from 160 human OS samples for chromosomal alterations around the *PTPRZ1* locus on chromosome 7q31.3, yet we did not detect any specific changes supporting a contribution of *PTPRZ1* loss to OS development or severity. We additionally analyzed exome sequencing data sets from 17 human OS samples, thereby identifying only one heterozygous germline mutation in the *PTPRZ1* gene, which did however not interfere with the anti-proliferative activity of the Rptpζ protein. We additionally searched the COSMIC database for mutations in PTP-encoding genes previously identified in human OS samples [63]. Consistent with the absence of published data reporting mutations or epigenetic silencing of *PTPRZ1* in OS cases, the only identified mutation in a PTP-encoding gene was not affecting *PTPRZ1*, but *PTPRT* (COSM1732508AA). Especially this latter observation led us to address the question, if *PTPRZ1* is differentially expressed in human osteoblasts, or if Rptpρ, encoded by the *PTPRT* gene, could be the most relevant phosphatase controlling proliferation of human osteoblasts. We therefore induced osteogenic differentiation of primary osteoblasts, SaOS-2 and U2-OS and found that *PTPRZ1* expression followed the same kinetic as observed in primary murine osteoblasts. In contrast, while *Ptprt* expression was barely and non-differentially expressed in murine cultures, *PTPRT* transcripts were undetectable in human osteoblasts. These data, together with the reduced proliferation of OS cell lines after transfection of a Rptpζ expression plasmid, imply that Rptpζ has a similar role in human and murine osteoblasts. Nevertheless, the absence of evidence supporting a role of Rptpζ as a human tumor suppressor gene, essentially demonstrates that it is not a major player in human OS development.

On the other hand, since there is evidence for *MDK* over-expression in human OS, it cannot be fully ruled out Rptpζ, non-mutated and normally expressed, is involved in OS development as a putative Mdk receptor. This is why it is potentially relevant that we were able to provide further evidence for a function of Mdk as an Rptpζ antagonist, by showing that the proliferative influence of Mdk on *Trp53* ^*+/-*^ OS cell lines was abrogated by *Ptprz1*-deficiency. In our opinion, it would additionally be useful to identify osteoblast-specific substrates dephosphorylated by Rptpζ and controlling proliferation. For now we did not succeed to address this question with a candidate approach, since we did not detect specific signals with antibodies against some phosphorylated proteins in *Ptprz1*-deficient OS cell lines. More specifically, the tested candidates included Fyn, Git-1 (both undetectable in the non-phosphorylated form), Src (pY416), Fak (pY397, pY567/577, pY925), and p42/44-Mapk (pT202/Y204). Therefore, it appears that the identification of osteoblast-specific Rptpζ downstream mediators requires utilization of unbiased approaches, such as substrate trapping. Even though there is no evidence so far supporting a role Rptpζ or its putative substrates in human OS development, it is quite relevant to state that Rptpζ is a transmembrane protein with restricted expression pattern. This implies that it is principally targetable for treatment, for instance by antagonizing its interaction with Mdk.

## Supporting Information

S1 Fig Skeletal analysis of *Trp53*-deficient mice. Contact Xrays and histological analysis of undecalcified spine or tibia sections from 12 weeks old mice of the indicated genotypes. No OS development was observed in either analysis.
(TIF)

S2 Fig Far Western-Blotting with additional SH2 domains. Shown are the profiles after probing the membranes with additional SH2 domains as indicated.
(TIF)

S1 Table Summary of OS development in individual mice. (PDF)
