# Supplementary figures and images for: The Protein Tyrosine Phosphatase Rptpζ Suppresses Osteosarcoma Development in Trp53-Heterozygous Mice
Source: PLoS One. 2015 Sep 11;10(9):e0137745. doi: 10.1371/journal.pone.0137745 (PMC4567063; doi:10.1371/journal.pone.0137745)

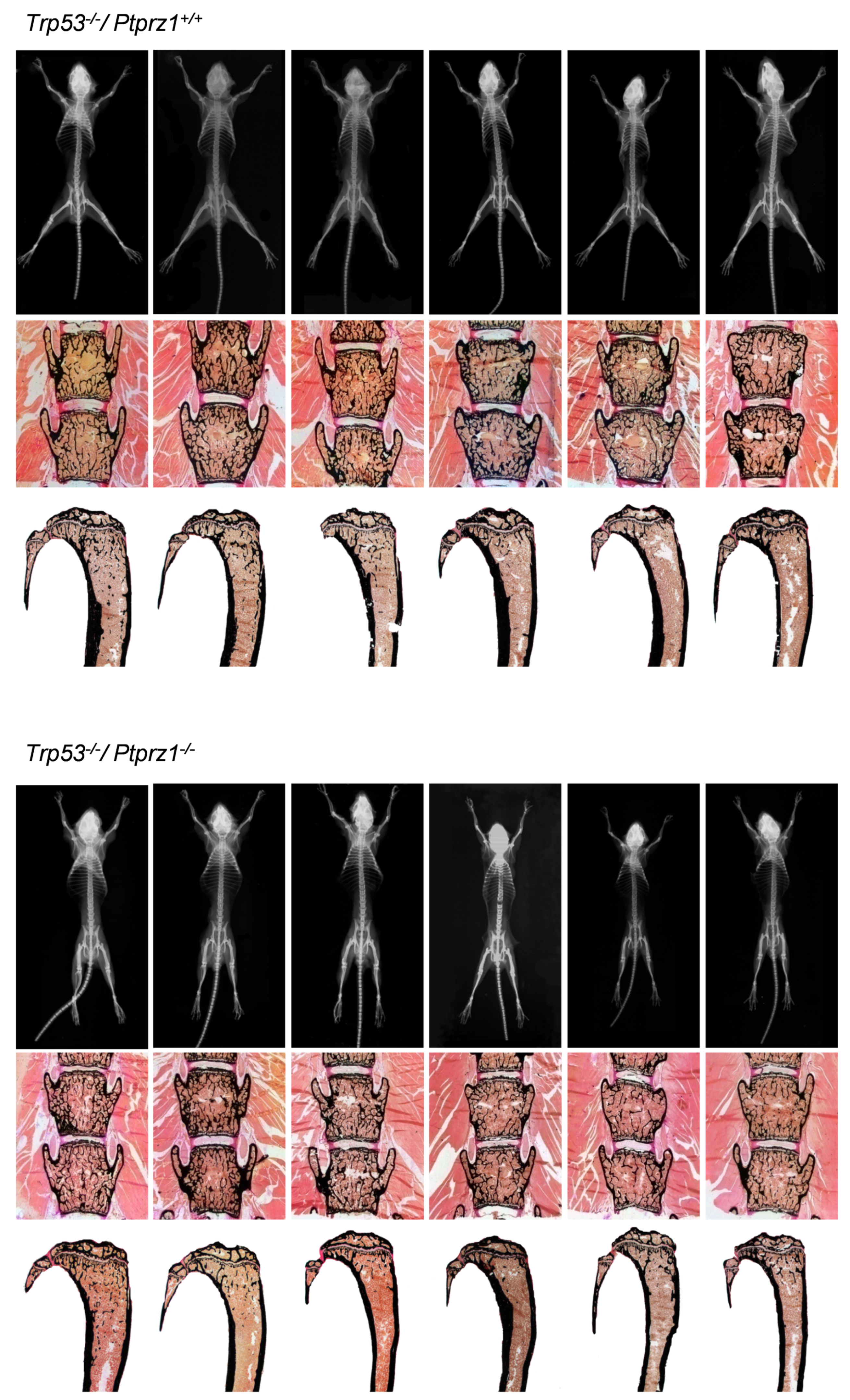

Supplement: S1 Fig — Contact Xrays and histological analysis of undecalcified spine or tibia sections from 12 weeks old mice of the indicated genotypes. No OS development was observed in either analysis. (TIF) [file pone.0137745.s001.tif]

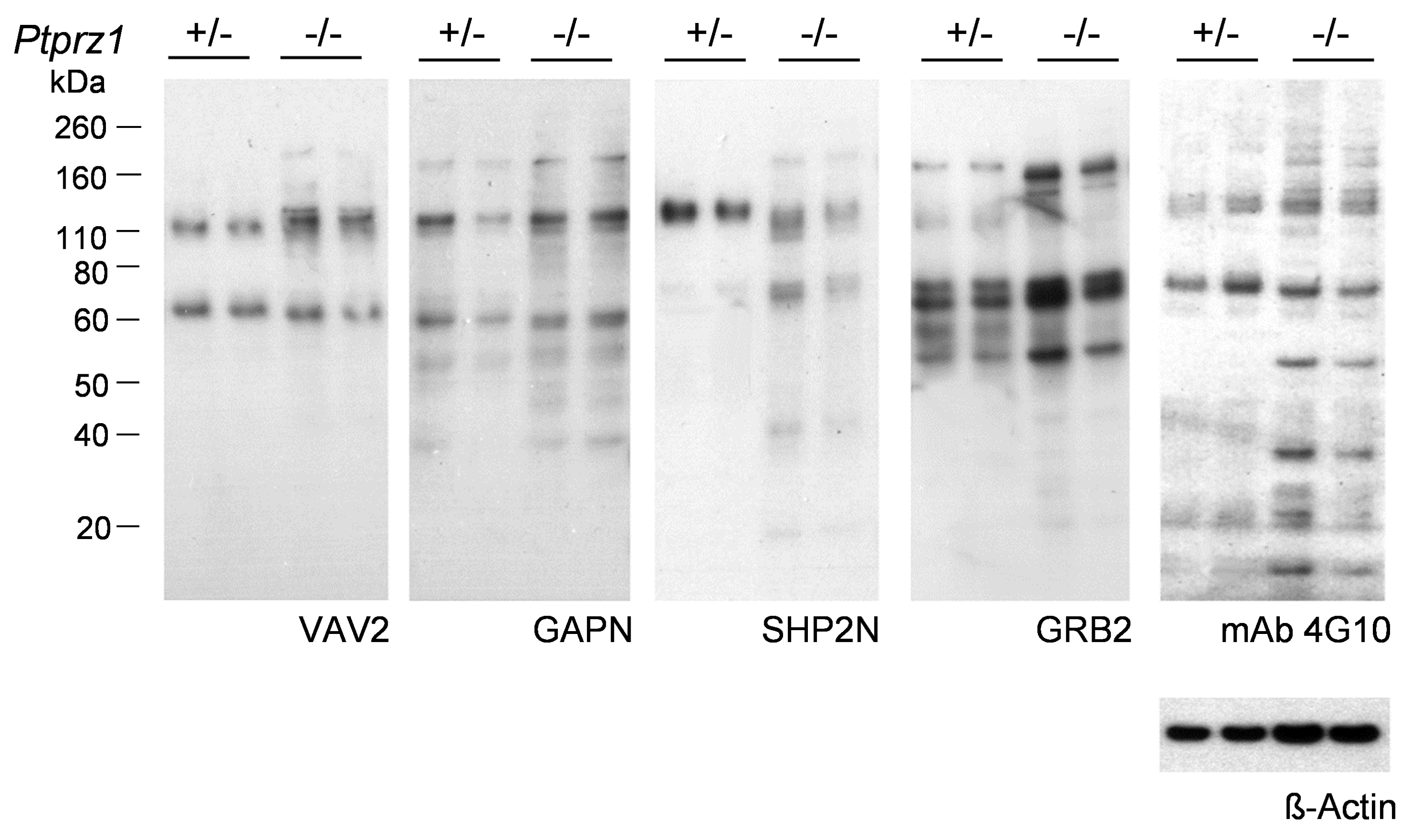

Supplement: S2 Fig — Shown are the profiles after probing the membranes with additional SH2 domains as indicated. (TIF) [file pone.0137745.s002.tif]
