# Supplementary material for: The Protein Tyrosine Phosphatase Rptpζ Suppresses Osteosarcoma Development in Trp53-Heterozygous Mice
Source: PLoS One. 2015 Sep 11;10(9):e0137745. doi: 10.1371/journal.pone.0137745 (PMC4567063; doi:10.1371/journal.pone.0137745)
Supplement: S1 Table — (PDF) [file pone.0137745.s003.pdf]

***Ptprz1* genotype**	**Gender**	**Age of OS detection**	**OS location**
**+/-**	female	52 wks	rib
**+/-**	female	29 wks	long bone (tibia)
**-/-**	male	52 wks	long bone (femur)
**-/-**	male	16 wks	rib
**-/-**	female	52 wks	spine (2 x)
**-/-**	female	52 wks	spine (3 x)
**-/-**	female	52 wks	long bone (femur)
**-/-**	female	47 wks	spine (2 x)
**-/-**	female	47 wks	spine
